# Pituitary *Neurolobectomy* induces sustained hypotension in male Wistar rats and normalizes blood pressure in male spontaneously hypertensive rats

**DOI:** 10.14814/phy2.70912

**Published:** 2026-05-29

**Authors:** Gloria Marcela Villanueva‐Rodríguez, Norma A. Bobadilla, J. Luis Quintanar, Claudia Verónica Rivera‐Cerecedo, David Roberto Chavira‐Ramírez, Kalman Kovacs, Andrés Quintanar‐Stephano

**Affiliations:** ^1^ Departamento de Fisiología y Farmacología. Centro de Ciencias Básicas Universidad Autónoma de Aguascalientes Aguascalientes México; ^2^ Departamento de Nefrología y Metabolismo Mineral Instituto Nacional de Ciencias Médicas y Nutrición, México, e Instituto de Investigaciones Biomédicas Universidad Nacional Autónoma de México Mexico City México; ^3^ Bioterio. Instituto de Fisiología Celular (IFC) Universidad Nacional Autónoma de México Mexico City México; ^4^ Departamento de Hormonas Esteroides Instituto Nacional de Ciencias Médicas y Nutrición Mexico City México; ^5^ Division of Pathology, Department of Laboratory Medicine St. Michael's Hospital Toronto Ontario Canada

**Keywords:** blood pressure, diabetes insipidus, hypertension, neurointermediate pituitary lobectomy, oxytocin, vasopressin

## Abstract

Arginine vasopressin is primarily recognized as an osmoregulatory hormone; however, its contribution to vascular tone and blood pressure regulation under basal and hypertensive conditions remains debated. This study examined the effects of vasopressin deficiency induced by neurointermediate pituitary lobectomy in Wistar and spontaneously hypertensive rats, compared with sham‐operated controls. Water intake, urine output, plasma vasopressin and oxytocin levels, blood pressure, and heart rate were evaluated over time. Neurointermediate pituitary lobectomy produced transient diabetes insipidus followed by the restoration of water balance, while plasma vasopressin and oxytocin levels remained markedly reduced at 3‐ and 90‐days post‐surgery (*p* < 0.0001 vs. Sham). This deficiency was associated with sustained hypotension in Wistar rats (~27 mmHg; *p* < 0.05) and normalized systolic blood pressure in spontaneously hypertensive rats (~50 mmHg; *p* < 0.001). Acute and chronic vasopressin and oxytocin deficiency causes transient diabetes insipidus, persistent hypotension in normotensive rats, and attenuated hypertension in spontaneously hypertensive rats with unchanged heart rate, demonstrating that vasopressin plays a key role in regulating both basal and hypertensive blood pressure.

## 1 INTRODUCTION

The hypothalamo‐neurohypophyseal system connects the hypothalamus to the posterior pituitary and consists of magnocellular neurons in the paraventricular (PVN) and supraoptic nuclei (SON) that synthesize arginine–vasopressin (AVP) and oxytocin (OT). These hormones are transported through the hypothalamo–neurohypophyseal tract to the neurohypophysis and released into the systemic circulation. In addition, parvocellular AVP‐ and OT‐producing neurons in the PVN project to extra‐hypothalamic regions (middle eminence, limbic system, and amygdale), where they regulate endocrine, emotional, and autonomic functions (Japundžić‐Žigon, 2013). Within the central nervous system, AVP and OT from parvocellular neurons also act as neurotransmitters and neuromodulators.

AVP exerts pleiotropic effects through three receptors (V1aR, V1bR, and V2R) located in peripheral organs and the CNS (Mavani et al., 2015). Its best‐known roles are the regulation of extracellular fluids and blood pressure (BP). Plasma hyperosmolality is sensed by osmoreceptors and stimulates AVP secretion, which promotes renal water reabsorption via the V2R–cAMP–aquaporin‐2 system in the principal cells of the collecting ducts, thereby restoring osmolality and concentrating urine (Schrier, 2008). Deficient AVP secretion causes central diabetes insipidus (DI), characterized by polyuria and polydipsia (Refardt et al., 2020).

The role of AVP in BP regulation is more complex. Through V1aR in vascular smooth muscle, AVP contributes to vasopressor responses, particularly under conditions such as hemorrhage or circulatory shock. Sensory inputs from baroreceptors, volume receptors, and chemoreceptors activate brainstem and PVN circuits that coordinate peripheral AVP release with sympathetic outflow to increase heart rate (HR), induce vasoconstriction, and restore BP (Aikins et al., 2021; Japundžić‐Žigon, 2013). However, in well‐hydrated, non‐stressed conditions, AVP does not appear to contribute significantly to basal BP maintenance, as shown in Brattleboro rats with a natural AVP gene deletion and in V1aR knockout mice, where BP is normal or only slightly reduced (Aikins et al., 2021; Japundžić‐Žigon et al., 2020; Koshimizu et al., 2012). This has been attributed to the fact that basal AVP concentrations (2–5 pg/mL in rats) are too low to activate the less sensitive V1a receptors, leaving vascular tone under the control of the renin–angiotensin II system and sympathetic mechanisms (Japundžić‐Žigon, 2013).

It is well established that AVP levels are elevated in essential hypertension in humans as well as in Spontaneously Hypertensive Rats (SHR) (Cowley Jr et al., 1981; Magnusson & Meyerson, 1996) and in Goldblatt models (one‐kidney, one‐clip (1K‐1C) and two‐kidney, one‐clip (2K‐1C) rats) (Woods & Johnston, 1982), however, its role in the regulation of basal BP remains unclear and controversial. This raises the question of how BP is affected in the absence or near absence of AVP. Some studies have reported a reduction in BP following administration of the V2 receptor antagonist OPC‐31260 in young male SHR in the prehypertensive phase (Verzicco et al., 2022). In contrast, in Dahl salt‐sensitive hypertensive rats, chronic treatment with a V1aR antagonist (OPC21268), a V2R antagonist (tolvaptan, TOLV), or their combination (OPC/TOLV) failed to modify BP during the study period (Ikeda et al., 2015). Overall, evidence regarding the effects of AVP deficiency on basal BP remains limited, conflicting, and inconclusive.

To determine whether AVP deficiency is required to maintain basal BP, the aim of this work was to study the effects of NIL on BP in normotensive rats in real time, acute, short, middle, and long‐term. In addition, HR, water intake, urine output, urinary and serum sodium levels, and plasma AVP and OT concentrations were assessed. Furthermore, in SHR, we assessed whether NIL‐induced AVP deficiency alters BP levels.

## 2 MATERIALS AND METHODS

### 2.1 Animals

Male Wistar rats, 3 months old (250–300 g body weight), were obtained from the UAA vivarium. Male SHR and their normotensive counterparts, Wistar‐Kyoto (WKY) rats, 5 months old, were obtained from the IFC‐UNAM vivarium. Animals were housed under controlled conditions: a 12/12 h light/dark cycle (lights on at 07:00 h), constant temperature (21°C ± 2°C), access to Lab Diet 5001 rodent chow (PMI Nutrition International, MN, USA) and water ad libitum. The number of rats used per experiment is reported in the corresponding figure and figure legends.

**FIGURE 1:**
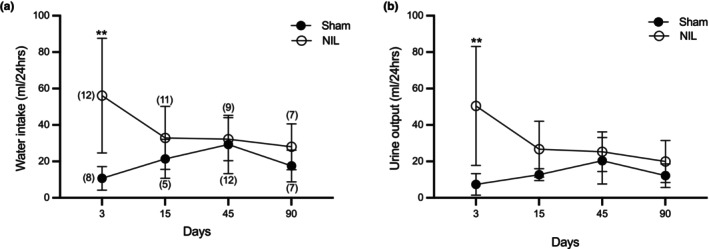
Effect of sham surgery and neurointermediate pituitary lobectomy (NIL) on water intake (a) and urine output (b) (mL/24 h). Animals were placed in metabolic cages at 3, 15, 45, and 90 days post‐surgery. NIL animals showed increased water intake and urine output during the early phase of exposure compared with Sham animals, with progressive normalization over time Data are presented as mean ± SD (*n* per group in parentheses). (a) Water intake: **p = 0.0015; (b) urine output: **p = 0.0031 versus sham at 3 days post‐NIL.

**FIGURE 2:**
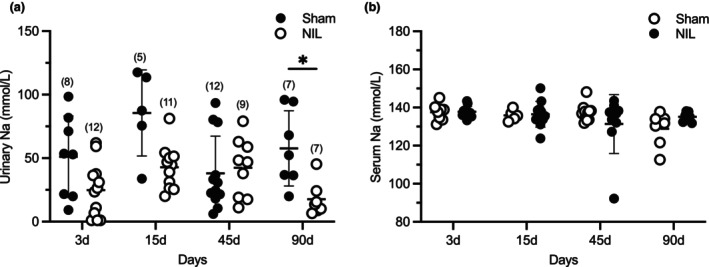
Effect of Sham surgery and NIL on urinary Na (a) and serum Na (b) at 3, 15, 45, and 90 days post‐surgery. A significant reduction in urinary sodium levels (a) was observed in NIL animals at 90 days compared to Sham controls. No significant differences between groups at any time point were observed in serum sodium levels (b) Data are expressed as mean ± SD (*n* per group in parentheses). (a) Urinary Na: *p = 0.0435 Sham versus NIL at 90 days post‐NIL.

**FIGURE 3:**
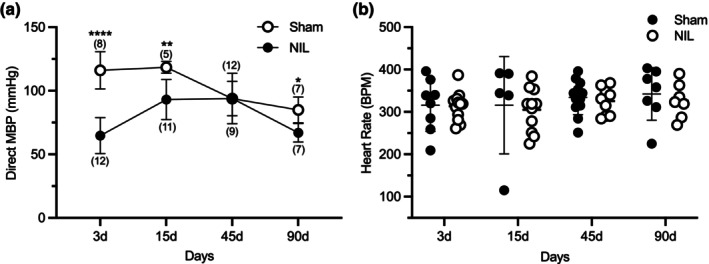
Effects of sham surgery and NIL on direct mean blood pressure (MBP) (a) and HR (b) at 3, 15, 45, and 90 days post‐surgery. MBP (a) was significantly lower in NIL animals compared to Sham controls at 3, 15, and 90 days. HR (b) was not significantly affected by NIL at any time point. Data are presented as mean ± SD (*n* per group in parentheses). *p = 0.0119, **p = 0.0011, ****p < 0.0001 versus respective sham controls.

To reach the study objectives, four experimental protocols were conducted:

Experiment 1. Evaluation of the effects of AVP deficiency at short (3 days), medium (15 days), and long‐term (45 and 90 days) post‐NIL on urine output and water intake (DI course), urinary and serum sodium levels, HR and basal arterial BP (Figures 1, 2, and 3).

**FIGURE 4:**
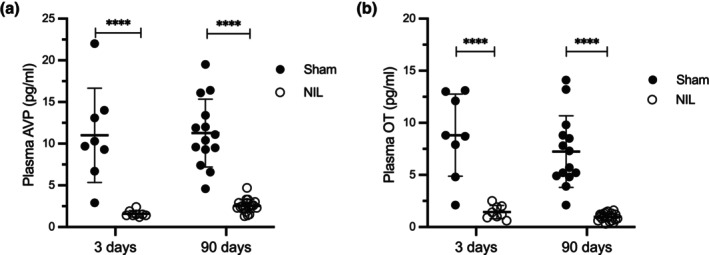
Acute (3 days) and long‐term (90 days) effects of Sham surgery and NIL on plasma levels of AVP (a) and OT (b). Different groups were prepared for each bleeding. AVP and OT levels were significantly lower in NIL animals than in Sham controls at both time points evaluated. Data are expressed as mean ± SD. ****p < 0.0001 Sham versus NIL at both times.

Experiment 2. Assessment of AVP and OT plasma levels at 3‐ and 90‐days post‐NIL (Figure 4).

**FIGURE 5:**
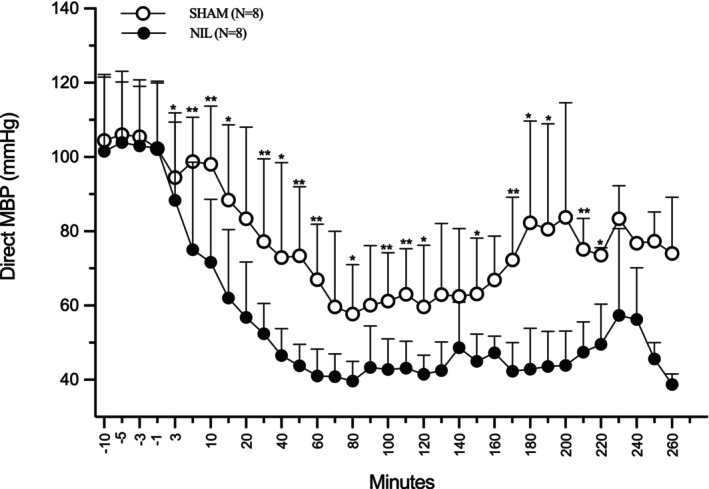
Acute effects of sham surgery and NIL on MBP. Before the surgery, the MBP basal records were taken at −10, −5, −3, and −1 min. The surgery was performed in real time and the MBP was measured again at 3, 5, 10, and every 10 min thereafter up to 260 min post‐surgery. A drop in pressure can be observed from minute 3 post‐surgery. Mean ± SD (*n* = 8 per group). *p < 0.05, **p < 0.01.

Experiment 3. Acute effects of AVP deficiency on arterial BP during the 260 min following NIL. Baseline BP was measured at −10, −5, −3, and −1 min before NIL. Following NIL or Sham surgery, BP was recorded at 1‐, 3‐, 5‐, and 10‐ min, and subsequently every 10 min until the end of the experiment at minute 260 (Figure 5).

**FIGURE 6:**
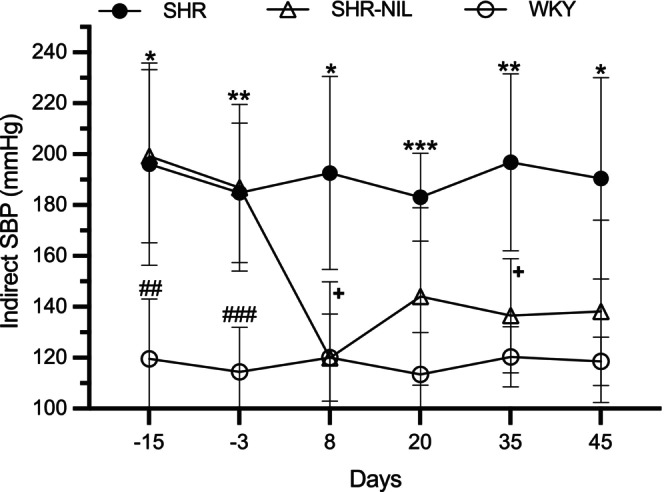
Effect of sham surgery and NIL on indirect SBP. Measurements were obtained at −15 and −3 days before surgery and at 8, 20, 35, and 45 days after NIL. Groups included SHR (*n* = 6), SHR–NIL (*n* = 8), and WKY (*n* = 5). Data are expressed as mean ± SD. *p* Values: *SHR versus WKY, + SHR versus SHR‐NIL, # SHR‐NIL versus WKY. *p = 0.0117 at −15 days, 0.0114 at 8 days, 0.0171 at 45 days. **p = 0.0021 at −3 days, 0.0060 at 35 days. ***p = 0.0003 at 20 days. +*p* = 0.0106 at 8 days, 0.0177 at 35 days. ##*p* = 0.0013 at −15 days and ###*p* = 0.0009 at −3 days.

Experiment 4. Evaluation of AVP deficiency on SBP using the noninvasive tail‐cuff sleeve method in SHR and their normotensive controls (WKY). Measurements were taken at 15‐ and 3‐ days prior to NIL, and subsequently at 8‐, 20‐, 35‐, and 45‐ days post‐NIL (Figure 6).

### 2.2 Neurointermediate pituitary lobectomy (NIL)

The pituitary gland was accessed using the parapharyngeal approach originally described by Serradeil‐Legal and Peters (Ben‐Jonathan & Peters, 1982), with minor modifications (Navarro‐Gonzalez et al., 2023). The surgical procedure was performed under a dissecting stereomicroscope (Zeiss OPMI‐19‐FC, 6× magnification).

In summary, rats were anesthetized with isoflurane (SofloranVet, Pisa Laboratories, Mexico), placed in the supine position, with the head and limbs fixed to the surgical table, and the trachea cannulated to ensure anesthesia and airway patency. A midline cervical incision (~2 cm) was made caudally from the skin tubercle of the beard. The digastric and pterygoid muscles were opened and surrounding tissues retracted, providing a broad surgical field that exposed the distal pterygoid process and the longus capitis muscles. The muscles were carefully scraped to expose the occipital bone, occipital crest, and the occipito‐sphenoidal joint, beneath which the pituitary gland is located. A 2 mm hole was drilled in the occipito‐sphenoidal joint to access the adenohypophysis. After removing bone debris, the dura mater was incised and the adenohypophysis gently elevated, allowing direct visualization of the neurohypophyseal lobe. The neurohypophysis was then carefully aspirated, removing the neurointermediate lobe. Minor bleeding occurred but ceased spontaneously. Retractors were removed and the incision was closed with two or three separate sutures. To prevent infection and minimize pain, a single intramuscular injection of Dexa‐Estreptovet 4500UI per 300 g (Agrovet Laboratories, Mexico.) was administered.

### 2.3 Sham NIL surgery

The procedure for the Sham surgery was identical to the NIL approach, except that it was interrupted once the occipito‐sphenoidal joint was clearly identified. Then, the skin incision was closed with sutures. To prevent infection and minimize pain, a single intramuscular injection of Dexa‐Estreptovet was administered.

### 2.4 Water intake and urine output assessments

Animals were individually housed in metabolic cages for 24 h, and water intake and urine output were recorded on days 3‐, 15‐, 45‐, and 90‐ following Sham or NIL surgery.

### 2.5 Urine and serum Na assessments

Sodium concentrations in urine and serum were determined using an automated analyzer (VITROS DT80, Ortho Clinical Diagnostics) based on dry chemistry technology. Measurements were performed by indirect potentiometry using sodium‐selective electrodes (ISE). Samples were diluted prior to analysis, and the electrical potential generated by sodium ions was converted to concentration according to the Nernst equation. Results were expressed in mmol/L.

### 2.6 Plasma AVP and OT assessments (ELISA)

Experiment 2 was conducted to evaluate the short‐ and long‐term effects of NIL and sham surgery on plasma AVP and OT levels. Separate groups of rats were used for the determination of AVP and OT concentrations at 3 and 90 days. To minimize hormone degradation associated with prolonged storage of plasma samples at −80°C, the long‐term (90‐day) NIL and sham groups were operated on 3 months prior to the corresponding short‐term (3‐day) groups. Consequently, the interval between blood collection from long‐ and short‐term groups did not exceed 15 days.

All animals were anesthetized with sodium pentobarbital (50 mg/kg body weight, i.p.) and blood samples were collected via the abdominal aorta. From each animal, 3 mL of blood were immediately drawn into ice‐chilled EDTA tubes and centrifuged at 1800 × g for 15 min at 4°C to obtain plasma. The plasma supernatant was then transferred to a new ice‐chilled tube and centrifuged again at 1800 × g for 10 min at 4°C to obtain platelet‐free plasma. Finally, plasma samples were aliquoted into 0.5 mL and stored at −80°C until AVP and OT assays were performed. On the day of the assay, plasma samples were thawed at room temperature and gently vortexed prior to loading onto ELISA plates. All samples were processed under standardized conditions to minimize pre‐analytical variability and all procedures were performed according to the manufacturer's instructions.

Plasma AVP concentrations were measured using a commercially available ELISA kit (Arginine Vasopressin ELISA Kit, Enzo Life Sciences, Cat. No. ADI‐901‐017, USA), with a sensitivity of 2.84 pg/mL and a linear detection range of 4.10–1000 pg/mL.

Plasma OT concentrations were measured using an ELISA kit (Oxytocin ELISA Kit, DGR Instruments, Cat. No. EIA‐5241, Germany), with a sensitivity of 11.7 pg/mL and a working range of approximately 15–1000 pg/mL. The assay demonstrated good reliability, with intra‐ and inter‐assay coefficients of variation below 10% and 12%, respectively.

### 2.7 Femoral arterial BP assessments

General description: After anesthesia induction, rats were placed on the surgical table, and the trachea was cannulated to ensure airway patency. A 2‐cm longitudinal incision was made below the midpoint of the inguinal fold of the left thigh. Under a stereomicroscope, the femoral artery was dissected and isolated; the distal end was ligated, and the proximal end was temporarily occluded. A small incision was made in the arterial wall, and a 10‐cm heparinized polyethylene catheter (PE‐10; Clay‐Adams) was inserted into the femoral artery. The catheter was secured and connected to a pressure transducer (TSD104A) coupled to an MP150 data acquisition system (BIOPAC Systems Inc.) running AcqKnowledge 4.1 software.

In experiment 1, animals were anesthetized with sodium pentobarbital (50 mg/kg b.w., i.p.). Femoral catheterization was performed as previously described. Direct MBP (mean blood pressure) and HR were calculated as the average of three consecutive measurements obtained in the same animal. Recordings were obtained for 15–20 min following femoral artery catheterization. Measurements were performed at 3‐, 15‐, 45‐, and 90‐days post‐NIL, using separate groups of rats at each time point (Figure 3a,b). Euthanasia was performed at the end of the procedure. The number of animals included in each group is indicated in parentheses in Figure 3a.

In experiment 3, anesthesia was induced with urethane (1.3 g/kg body weight, intraperitoneally; Sigma, Cat. U2500, USA). To prevent asphyxia during NIL or Sham surgery, the trachea was cannulated prior to the procedure. Following femoral catheterization, baseline MBP was recorded at −10, −5, −3, and −1 min before surgery, which was completed within approximately 6 min. Immediately after surgery, MBP was measured at 3, 5, 10, 20, and at 10‐min intervals thereafter, up to 260 min (Figure 5).

### 2.8 Indirect systolic arterial BP assessment

Systolic blood pressure (SBP) in SHR and normotensive WKY rats was measured noninvasively using the tail‐cuff method. Measurements were obtained using a BP transducer (TSD104A) connected to a DA100C amplifier and a pulse transducer (TSD200) connected to a PPG100C amplifier. Both signals were interfaced through a Universal Interface Module to an MP150 data acquisition system. Data were recorded and analyzed using AcqKnowledge software (version 4.1; BIOPAC Systems Inc., USA).

Prior to BP measurements, animals were acclimated to the procedure by daily exposure to the immobilization chamber for 2 weeks (5 days per week, Monday–Friday). Animals were placed in the chamber without physical restraint. Training sessions were conducted each day at 9:00 a.m., maintaining a consistent order of animals to reduce variability. To ensure optimal recording conditions, the chamber was placed on a thermal platform to maintain a temperature of 35°C. Each rat was allowed to stabilize in the chamber for 10–15 min prior to measurement. The occlusion cuff was positioned on the proximal third of the tail, and the pulse transducer was placed on the middle third, immediately distal to the cuff. Following stabilization, SBP was recorded over a 15–20‐min period per animal. During this time, at least three consecutive measurements were obtained, and the mean SBP was calculated.

### 2.9 Sacrifice

Animals were euthanized by an overdose of sodium pentobarbital (200 mg/kg b.w.). Postmortem stereomicroscopic examination (6× magnification) confirmed the integrity of the adenohypophysis and the extent of the NIL. A consistent regeneration of a small neurohypophyseal lobe was observed in NIL rats, originating from the proximal pituitary stalk, whereas in Sham rats both the adenohypophyseal and neurohypophyseal lobes remained normal. Animals that did not meet these criteria were excluded from the analysis.

### 2.10 Statistical analysis

Multiple comparison tests between NIL and Sham groups from the four experiments were performed using a two‐way ANOVA followed by Sidak's post hoc test. For variables involving continuous repeated measurements (minute‐by‐minute BP), linear mixed‐effects models (REML) were applied. All analyses were performed using GraphPad Prism software version 10.0 (GraphPad Software, Inc., CA, USA). Differences between groups were considered statistically significant at *p* < 0.05.

### 2.11 Ethical statement

All applicable institutional and national guidelines for the care and use of animals were followed. Animal procedures were supervised by the Animal Care and Use Committee of the Universidad Autónoma de Aguascalientes (UAA), in compliance with CEADI‐UAA regulations (approval code CEADI‐UAA/06/2025). These regulations are in strict accordance with the Mexican Official Standard NOM‐062‐ZOO‐1999, which provides the technical specifications for the production, care, and use of laboratory animals. At the end of the experiments, animals were euthanized by an overdose of sodium pentobarbital administered intraperitoneally or intra‐arterially. Every effort was made to minimize animal suffering throughout all experimental procedures.

## 3 RESULTS

Figure 1 shows the effects of Sham and NIL surgeries on water intake (a) and urine output (b) for 24 h at 3‐, 15‐, 45‐ and 90‐days post‐surgery. NIL animals exhibited higher water intake compared to sham controls (37.3 ± 17.4 vs. 19.7 ± 10.8 mL/day; mean difference: −17.6 mL, 95% CI: −26.4 to −8.8). Post hoc analysis (Šídák's test) demonstrated that this difference was significant at 3 days (56.1 ± 31.7 vs. 10.7 ± 6.1 mL/day, *p* = 0.0015), but not at later time points (15 days: 32.9 ± 17.4 vs. 21.4 ± 9.8; 45 days: 32.2 ± 11.0 vs. 29.3 ± 16.7; 90 days: 28.1 ± 12.5 vs. 17.5 ± 8.8 mL/day; *p* > 0.05 for all). Urine output was significantly higher in NIL animals compared to sham controls at 3 days (50.5 ± 32.7 vs. 7.4 ± 5.7 mL/day, mean difference: −43.1 mL, *p* = 0.0031). A trend toward higher urine output was observed at 15 days (*p* = 0.0537), but no significant differences were found at 45 or 90 days (*p* > 0.05). These findings indicate a transient increase in water intake and urine output following NIL, with a progressive normalization over time.

Figure 2 shows the effects of Sham and NIL surgeries on urinary Na (a) and serum Na (b) at 3‐, 15‐, 45‐, and 90‐days post‐surgery. The Figure 2a urinary sodium excretion shows no significant differences between groups at 3 days (Sham: 51.09 ± 30.5 vs. NIL: 24.77 ± 20.5), 15 days (Sham: 85.56 ± 33.0 vs. NIL: 42.80 ± 18.5), or 45 days (Sham: 38.03 ± 28.0 vs. NIL: 42.39 ± 22.0). However, a significant reduction in urinary sodium levels was observed in NIL animals at 90 days compared to sham controls (Sham: 57.69 ± 28.0 vs. NIL: 17.74 ± 13.0; *p* = 0.0435).

Serum sodium concentrations revealed no significant effects of time [*F* (2.031, 27.76) = 1.807, *p* = 0.1826], treatment (Sham vs. NIL; *F* (1, 22) = 0.0145, *p* = 0.9052), or time × treatment interaction (*F* (3, 41) = 2.119, *p* = 0.1125). Post hoc analysis confirmed the absence of differences between groups at all time points (3‐, 15‐, 45‐, and 90 days; all *p* > 0.05).

These results indicate that systemic sodium levels remained stable throughout the experimental period, despite the reduction in urinary sodium excretion observed in NIL animals.

Figure 4 shows the acute (3 days) and long‐term (90 days) effects of Sham and NIL surgeries on AVP and OT plasma levels. In the Figure 4a AVP levels were significantly lower in NIL animals compared to sham controls at both time points evaluated. At 3 days, AVP levels were 10.85 ± 5.6 pg/mL in sham animals and 1.70 ± 0.36 pg/mL in NIL animals (mean difference: 9.15 pg/mL, *p* < 0.0001), and this difference persisted at 90 days (11.27 ± 4.2 vs. 2.53 ± 0.80 pg/mL, mean difference: 8.75 pg/mL, p < 0.0001). In the Figure 4b at 3 days, OT levels were 8.81 ± 3.89 pg/mL in sham animals and 1.44 ± 0.63 pg/mL in NIL animals (mean difference: 7.38 pg/mL, *p* < 0.0001), and this difference persisted at 90 days (7.24 ± 3.49 vs. 0.97 ± 0.36 pg/mL, respectively; mean difference: 6.27 pg/mL, *p* < 0.0001). Thus, circulating AVP and OT levels were lower following NIL, with no evidence of recovery.

Figure 3, shows the effects of Sham and NIL surgeries on direct MBP (a) and HR (b) at short (days 3), middle (15 days), and long‐term (45 and 90 days); values are presented as mean ± SD. MBP was significantly lower in NIL animals compared to sham controls at 3 days (64.7 ± 14.2 vs. 116.1 ± 14.6 mmHg, mean difference: 51.4 mmHg, *p* < 0.0001), 15 days (93.0 ± 15.7 vs. 118.4 ± 4.6 mmHg, mean difference: 25.4 mmHg, *p* = 0.0011), and 90 days (66.9 ± 7.3 vs. 84.9 ± 10.2 mmHg, mean difference: 18.0 mmHg, *p* = 0.0119). No significant differences were observed at 45 days (93.9 ± 19.8 vs. 93.8 ± 13.5 mmHg, *p* > 0.9999).

NIL animals exhibited a marked reduction in arterial BP compared to sham controls, with an average difference of 23.6 mmHg (95% CI: 15.95–31.22).

HR was not significantly affected by NIL at any time point. Mixed‐effects analysis revealed no significant effect of time [*F* (2.298, 31.41) = 0.77, *p* = 0.4893], treatment (Sham vs. NIL; *F* (1, 22) = 0.47, *p* = 0.5022), or time × treatment interaction (*F* (3, 41) = 0.047, *p* = 0.9861).

Consistent with these findings, Šídák's multiple comparisons test showed no significant differences between groups at any time point (3‐, 15‐, 45‐, and 90 days; all *p* > 0.97).

Notably, HR remained unchanged despite the marked reduction in arterial BP.

Figure 5 shows the acute effects of Sham and NIL surgeries on MBP. Data are expressed as mean ± SD. Direct MBP was recorded at −10, −5, −3, and −1 min before NIL and Sham surgeries, and subsequently at 3, 5, 10, 20, and every 10 min thereafter up to 260 min post‐surgery. A linear mixed‐effects statistic model revealed significant differences between NIL and Sham groups beginning at minute 10 post NIL. *p < 0.05, **p < 0.01, and ***p < 0.001 indicate significant differences between the groups at each time point. Compared with Sham animals, NIL surgery produced a faster and more pronounced drop in MBP, reaching a nadir of approximately 45 mmHg around minute 40, and remaining around these low BP levels until the end of the experiment. A mild but nonsignificant rise in MBP was observed between minutes 210 and 240, followed by a return at minutes 250 and 260, respectively. Statistical differences between Sham and NIL groups occurred at minutes 10, 30, 40, 50, 60, 70, 80, 90, 100, 110, 150, 160, and 170. Overall, arterial BP was lower in NIL animals compared to sham controls (55.91 ± 9.44 vs. 77.16 ± 9.44 mmHg; mean difference: −21.25 mmHg, 95% CI: −31.91 to −10.60). However, in both groups of animals from minute 170 onward, the sample size (*n*) gradually decreased (rats start dying), resulting in smaller group sizes and the loss of statistical significance. The results provide direct evidence for a real‐time hemodynamic role of AVP.

Figure 6 shows the SBP values in SHR, SHR–NIL, and WKY rats measured at −15 and −3 days (before NIL surgery) and at 8, 20, 35, and 45 days after NIL surgery. Intact SHR and normotensive WKY rats were used as controls for comparison with the SHR–NIL group. Significant differences were not found between SHR and SHR‐NIL groups before surgery (−15 and −3 days), with mean ± SD values of 196.0 ± 37.55 vs. 199.2 ± 12.44 mmHg at −15 days and 184.8 ± 28.91 vs. 186.8 ± 10.25 mmHg at −3 days, respectively. In contrast, both groups exhibited significantly higher BP compared to WKY (119.5 ± 54.50 mmHg at −15 days and 114.4 ± 47.92 mmHg at −3 days).

Following NIL, significant differences between SHR and SHR‐NIL animals emerged at several time points, including day 8 (192.6 ± 40.95 vs. 119.9 ± 10.88 mmHg) and day 35 (196.8 ± 21.80 vs. 136.5 ± 7.99 mmHg; *p* < 0.05). Notably, no significant differences were observed between SHR‐NIL and WKY at any post‐surgical time point, with comparable mean ± SD values at day 8 (119.9 ± 10.88 vs. 120.1 ± 25.37 mmHg), day 20 (144.1 ± 7.35 vs. 113.4 ± 44.67 mmHg), day 35 (136.5 ± 7.99 vs. 120.4 ± 23.52 mmHg), and day 45 (138.2 ± 15.92 vs. 118.5 ± 37.48 mmHg).

In contrast, SHR animals maintained significantly higher BP than WKY rats throughout the experimental period, with mean ± SD values remaining elevated at day 8 (192.6 ± 40.95 vs. 120.1 ± 25.37 mmHg), day 20 (183.1 ± 18.01 vs. 113.4 ± 44.67 mmHg), day 35 (196.8 ± 21.80 vs. 120.4 ± 23.52 mmHg), and day 45 (190.5 ± 42.56 vs. 118.5 ± 37.48 mmHg) (*p* values in the Figure 6).

## 4 DISCUSSION

Our experiments demonstrate that permanent AVP deficiency induces a sustained and significant reduction in blood pressure (BP), while largely preserving overall balance (Figure 1). Urinary sodium excretion exhibited a nonsignificant decrease at 3, 15, and 45 days post‐NIL compared with Sham controls, reaching statistical significance only at day 90 (*p* = 0.0435). In contrast, serum sodium concentrations remained unchanged between NIL and Sham groups across all time points. These findings suggest the presence of compensatory renal mechanisms that preserve systemic sodium balance despite reduced AVP levels providing a consistent physiological framework supporting that the observed hemodynamic effects are primarily related to AVP deficiency rather than to secondary changes in fluid or sodium balance (Figure 2a,b).

In both normally hydrated rats and humans, the regulation of extracellular fluids is maintained by low circulating concentrations of AVP (2–5 pg/mL in rats and 1–5 pg/mL in humans) (Japundžić‐Žigon, 2013; Koshimizu et al., 2012). In NIL rats, AVP and OT plasma levels were permanently reduced (~80% and ~90% below the respective Sham groups), suggesting that the low basal AVP plasma levels from the remnant and reorganized pituitary stalk of the hypothalamic–neurohypophyseal fibers may contribute to the resolution of DI around day 15 post‐NIL, as was previously described by Moll and de Wied (1962). It should be noted that several AVP and OT concentrations in the NIL groups fell below the lower limit of detection (<2.84 pg/mL for AVP and <11.7 pg/mL for OT) and were therefore estimated by extrapolation. This approach yielded mean concentrations of 1.98 ± 0.43 pg/mL for AVP and 1.21 ± 0.51 pg/mL for OT (mean ± SD). Even though measurements within this extrapolated range may exhibit increased variability (approximately 30%–50% coefficient of variation), the results consistently indicate that AVP and OT levels were markedly reduced.

Although low basal AVP concentrations are sufficient to restore DI in NIL animals, present results exhibited a sustained reduction in arterial BP levels. This may partially be explained by the fact that the vascular V1a receptors require AVP serum levels around 300 pg/mL to induce vascular constriction as was reported by Japundžić‐Žigon (2013) and Thibonnier et al. (1998). In Brattleboro rats, arterial BP remains barely within normal ranges (Aikins et al., 2021; Japundžić‐Žigon et al., 2020; Koshimizu et al., 2012), suggesting that AVP plays only a minor role in BP regulation, which is largely maintained by sympathetic and renin–angiotensin mechanisms (Japundžić‐Žigon, 2013). In contrast, Simon et al. (1984), reported that hypophysectomy caused chronic hypotension, and that it is only partially compensated by the renin–angiotensin system. Similarly, mutant mice lacking the V1a receptor gene (V1a−/−) exhibited lower basal BP compared with wild‐type controls (Aikins et al., 2021; Koshimizu et al., 2006), this finding supports our view that AVP is required to maintain the basal BP levels. Although it is well recognized that sodium pentobarbital, the anesthetic used in the experiment 1, exerts hypotensive effects by depressing central sympathetic outflow and reducing vascular tone (Maggi & Meli, 1986). This effect, however, was present in both Sham and NIL groups. Despite this background influence, the reduction in MBP was important in NIL rats compared to their respective Sham groups (as shown in Figure 3a). This strongly suggests that the additional fall in BP is attributable to AVP deficiency rather than to the anesthetic itself. Moreover, whereas sympathetic mechanisms and the renin–angiotensin system are usually sufficient to maintain BP under non‐stressful conditions, the important and permanent hypotension observed in NIL rats indicates that AVP provides an additional vasopressor tone under basal circumstances. This is particularly relevant considering that there are other potent vasoconstrictors, such as angiotensin II, endothelin‐1, catecholamines, which remain active in these animals, indicating that they cannot fully compensate for the absence of AVP. These results reinforce the view that AVP is not only critical for acute decreases in BP, such as hemorrhage, but also contributes significantly to the maintenance of basal vascular resistance and arterial BP.

Crofton et al. (1979) demonstrated that Brattleboro rats failed to develop hypertension after deoxycorticosterone‐salt treatment, a standard model for experimental hypertension. Similarly, in our experiment 4, SHRs subjected to NIL showed a marked reduction in SBP, reaching levels comparable to those of normotensive WKY controls (Figure 6). This normalization was sustained throughout the 45‐day duration of the experiment. Supporting this notion, in DOCA‐salt hypertensive rats, blockade of V1a receptors with the selective antagonist OPC‐21268 produced a significant reduction in MBP (Burrell et al., 1994). Together with our results, these findings highlight the importance of AVP in its potential contribution to the pathophysiology of hypertension.

HR were obtained from the same animals used for direct MBP recordings (Experiment 1, Figure 3b). Comparison between the sham and NIL groups showed no significant differences in HR across the different time points evaluated in the experiment. AVP has been shown to play a key role in cardiovascular regulation by modulating both vascular tone and autonomic function. In addition to its well‐known vasoconstrictor effects, AVP enhances baroreflex sensitivity in area postrema and influences HR through both peripheral and central mechanisms, including actions within the nucleus tractus solitarius (Cowley Jr et al., 1974; Japundžić‐Žigon et al., 2020; Michelini & Bonagamba, 1988; Savić et al., 2022). Furthermore, impaired baroreflex function has been described in AVP‐deficient models such as Brattleboro rats (Zerbe et al., 1982). In this context, the absence of HR changes in our study despite a marked and sustained reduction in MBP strongly suggests a disruption of normal baroreflex‐mediated compensatory responses. Under physiological conditions, hypotension is expected to trigger a robust increase in HR via sympathetic activation; however, this response was not observed following NIL. AVP deficiency leads to sustained hypotension without appropriate HR compensation. These findings support the notion that endogenous AVP is required not only for maintaining vascular tone but also for preserving effective autonomic cardiovascular regulation.

Although plasma AVP levels were not measured in SHR‐NIL rats, we infer that they were reduced, as indicated by the permanent normalization of arterial BP and the small neurohypophyseal remnant lobe at necropsy due to pituitary stalk reorganization. A total of seven NIL rats were excluded from all experiments because they showed evidence of incomplete resection of the neural lobe, complete absence of the pituitary gland, or signs of adenohypophysis damage.

This work reintroduces an old but insufficiently studied experimental model of AVP deficiency—the NIL rat. Using this model, we demonstrate that AVP is a contributing factor in the maintenance of peripheral vascular resistance and basal vascular tone under both normotensive and hypertensive conditions, suggesting that the long‐standing claim that AVP does not contribute to basal BP regulation should be reconsidered. At the same time, our findings reinforce the classic concept that a minimal effective AVP concentration is required to preserve water balance.

Finally, the NIL rat model provides a valuable tool for further exploring the physiological and pathophysiological roles of AVP and OT, not only in cardiovascular regulation and extracellular fluid balance, but also across other organ systems expressing AVP receptors.

## 5 CONCLUSION

Our findings demonstrate that NIL induces a sustained reduction in circulating AVP and OT levels, transient DI, and a rapid and persistent decrease in arterial BP in both normotensive and hypertensive rats, while the HR remains unchanged, independently of fluid balance, supporting a direct role of AVP in the regulation of arterial pressure.

## 6 CLINICAL RELEVANCE

The present study demonstrates that AVP exerts a sustained and direct influence on BP regulation. The hypotension observed in NIL rats and the normalization of SBP in hypertensive rats indicate that AVP contributes both to the maintenance of basal vascular tone and to the pathophysiology of hypertension. From a clinical perspective, understanding the dynamics of AVP‐mediated vasoregulation could guide the development of new therapeutic strategies targeting AVP receptors (V1a and V2) in cardiovascular and neuroendocrine disorders.

## AUTHOR CONTRIBUTIONS

**Gloria Marcela Villanueva‐Rodríguez:** Conceptualization; data curation; formal analysis; funding acquisition; investigation; methodology; project administration; resources; supervision; validation; visualization. **Norma A. Bobadilla:** Formal analysis; methodology; visualization. **J. Luis Quintanar:** Methodology; visualization. **Claudia Verónica Rivera‐Cerecedo:** Investigation; methodology; project administration; resources; validation. **David Roberto Chavira‐Ramírez:** Data curation; investigation; methodology; project administration; resources; validation; visualization. **Kalman Kovacs:** Data curation; formal analysis; investigation; methodology; supervision; validation; visualization. **Andrés Quintanar‐Stephano:** Conceptualization; data curation; formal analysis; funding acquisition; investigation; methodology; project administration; resources; supervision; validation; visualization.

## FUNDING INFORMATION

This project was supported by UAA‐PIFF14‐1 (AQS) and CONACyT‐221262 (AQS) grants (México). GMVR was supported by CONACyT for her PhD scholarship (CVU: 452329).

## CONFLICT OF INTEREST STATEMENT

The authors declare no competing interests.

## CODE AVAILABILITY

No custom code was developed for this study. Data analyses were performed using commercially available software (GraphPad Prism software version 10.0).

## ETHICS STATEMENT

All applicable institutional and national guidelines for the care and use of animals were followed. Animal procedures were supervised by the Animal Care and Use Committee of the Universidad Autónoma de Aguascalientes (UAA), in compliance with CEADI‐UAA regulations (approval code CEADI‐UAA/06/2025). These regulations are in strict accordance with the Mexican Official Standard NOM‐062‐ZOO‐1999, which provides the technical specifications for the production, care, and use of laboratory animals. At the end of the experiments, animals were euthanized by an overdose of sodium pentobarbital administered intraperitoneally or intra‐arterially. Every effort was made to minimize animal suffering throughout all experimental procedures.

## Data Availability

All data supporting the findings of this study are available within the article and in the accompanying figure source data files.
